# Protocol for the IDEAL-2 longitudinal study: following the experiences of people with dementia and their primary carers to understand what contributes to living well with dementia and enhances active life

**DOI:** 10.1186/s12889-018-6129-7

**Published:** 2018-10-30

**Authors:** Barbora Silarova, Sharon M. Nelis, Rosalie M. Ashworth, Clive Ballard, Marta Bieńkiewicz, Catherine Henderson, Alexandra Hillman, John V. Hindle, Julian C. Hughes, Ruth A. Lamont, Rachael Litherland, Ian R. Jones, Roy W. Jones, Martin Knapp, Piers Kotting, Anthony Martyr, Fiona E. Matthews, Robin G. Morris, Catherine Quinn, Jemma Regan, Jennifer M. Rusted, Eleanor Ann van den Heuvel, Christina R. Victor, Yu-Tzu Wu, Linda Clare

**Affiliations:** 1 Centre for Research in Ageing and Cognitive Health (REACH), College of Medicine and Health, South Cloisters, St Luke’s Campus, Exeter, EX1 2LU UK; 2 Alzheimer’s Society Centre of Excellence, Centre for Research in Ageing and Cognitive Health (REACH), College of Medicine and Health, South Cloisters, St Luke’s Campus, Exeter, EX1 2LU UK; 3 College of Medicine and Health, St Luke’s Campus, Exeter, EX1 2LU UK; 4 London School of Economics and Political Science, Houghton Street, London, WC2A 2AE UK; 5 WISERD, Cardiff University, 38 Park Place, Cardiff, CF10 3BB UK; 6 RICE (The Research Institute for the Care of Older People), Royal United Hospital, Bath, BA1 3NG UK; 7 University of Bristol, Department of Population and Health Sciences, Bristol Medical School, Bristol, BS8 2BN UK; 8 Innovations in Dementia, PO Box 616, Exeter, EX1 9JB UK; 9 Newcastle University, Institute for Health and Society, Biomedical Research Building, Campus for Ageing and Vitality, Newcastle upon Tyne, NE4 5PL UK; 10 King’s College London, Henry Wellcome Building, Institute of Psychiatry, Psychology, and Neuroscience, De Crespigny Park, London, SE5 8AF UK; 11 University of Sussex, School of Psychology, Pevensey 1 2B21, Falmer, Brighton, BN1 9QH UK; 12 Brunel University London, College of Health and Life Sciences, Department of Clinical Sciences, Kingston Lane, Uxbridge, UB8 3PH UK

**Keywords:** Alzheimer’s disease, Caregivers, Dementia, Longitudinal studies, Quality of life

## Abstract

**Background:**

There is a major need for longitudinal research examining the experiences of people with dementia and their primary carers, as relatively little is known about how the factors associated with capability to ‘live well’ vary over time. The main aim of the IDEAL-2 study is to investigate how and why, over time, people with dementia and their primary carers might vary in their capability to live well with dementia, whilst exploring both their use of health and care services and their unmet needs.

**Methods:**

IDEAL-2 will build on the Improving the experience of Dementia and Enhancing Active Life (IDEAL) cohort of 1547 people (who, at recruitment between July 2014 and July 2016, had mild-to-moderate dementia), and their 1283 primary carers in Great Britain. The existing cohort will be enriched with additional participants with mild-to-moderate dementia (and their primary carers where available and willing) from the following groups: people with rarer forms of dementia, and/or those who are ≥90 years or < 65 years of age at time of recruitment. We will assess the primary outcome, capability to live well with dementia, and the factors influencing it using questionnaires at yearly intervals for 3 years. Additionally, we will seek to link the cohort data with administrative data to obtain information about health service use. Some participants will be invited for in-depth face-to-face interviews. The cohort study will be supplemented by linked research focusing on: the co-production of new measures of living well; including the perspectives of people with advanced dementia living in residential care settings; including people with dementia from black, Asian, and minority ethnic groups; and understanding the experience of people living with undiagnosed dementia.

**Discussion:**

IDEAL-2 will provide evidence about the key indicators of, and factors associated with, living well over the course of dementia and how these differ for particular subgroups. It will tell us which combinations of services and support are most beneficial and cost-effective. Moreover, the IDEAL-2 study will gather evidence from under-researched groups of people with dementia, who are likely to have their own distinct perceptions of living well.

**Electronic supplementary material:**

The online version of this article (10.1186/s12889-018-6129-7) contains supplementary material, which is available to authorized users.

## Background

Relatively few intervention trials have demonstrated benefits in terms of improving quality of life and well-being for people with dementia [1, 2], and we still know relatively little about how to improve the experience of people with dementia and their primary carers (e.g. relatives/friends) in real-world contexts. If interventions and initiatives that genuinely enhance this experience are to be developed, we first need a full understanding of the wide range of factors that influence whether people with all types of dementia can ‘live well’ with the condition, including people from black, Asian and minority ethnic (BAME) groups, and people with advanced dementia.

Researchers and members of the general public have different understandings of what the concept of living well actually means. In the literature, the concept of living well with dementia is usually operationalised in terms of good quality of life or health-related quality of life [3]. In our previous work [4] we have developed a conceptual framework for understanding living well that emphasises that the capability to live well with dementia is broader than quality of life [5] and includes well-being [6] and satisfaction with life [7]. We have also postulated that the capability to live well with dementia is shaped by several key factors: capitals, assets, and resources; challenges; access to and use of services; and the degree of adaptation achieved by the person and family [4]. This framework will continue to shape the development of our theoretical work throughout the IDEAL-2 study: *Improving the experience of Dementia and Enhancing active Life: a longitudinal perspective on living well with dementia*.

Importantly, people with dementia also have their own understanding of living well with dementia, but standard questionnaires do not always fully capture the most relevant aspects for each individual. One reason for this is that involvement of people with dementia in the development of living well measures has not yet extended to genuine co-production [8] where people with dementia work on equal terms with researchers to develop a scale that reflects their experiences and needs, something that researchers increasingly acknowledge as important [9, 10]. The construction of a new measure of the broader construct of living well in the IDEAL-2 study presents an important opportunity for people living with dementia to work together with the project team to co-produce a measure.

The IDEAL-2 study will also gather evidence from under-researched groups of people with dementia, who are likely to have their own distinct perceptions of living well [11]: people with advanced dementia living in residential care settings, people from BAME communities, and people who meet criteria for a diagnosis of dementia but have not accessed specialist services. More specifically, individuals with advanced dementia represent a group that is largely neglected in research. Little is known about their quality of life and evidence-based psychosocial interventions designed to meet their needs are almost entirely lacking [12–14]. Yet many people with advanced dementia are able to say what is important to them [11, 15], while others can communicate their feelings and preferences non-verbally [16, 17]. More effort is needed to obtain their views and include them in research, and in the IDEAL-2 study we aim to develop a communication toolkit to achieve this.

Next, evidence indicates that there is increased prevalence of dementia in people of African-Caribbean and South Asian ethnicity living in the United Kingdom (UK) [18, 19]. We know that these groups are less likely to access health services [20], leading to low diagnosis rates, and that they are at risk of lower well-being, for example due to high levels of loneliness [21]. In the IDEAL-2 study, we aim to identify how we can best understand the needs of people with dementia from BAME communities and ensure that their voices are represented in research and policy making. We will investigate ways to ensure that services provided by the National Health Service (NHS) are accessible and provide appropriate support to BAME communities. Levels of family care provision are also significantly higher in the African-Caribbean and South Asian groups in Britain than for other groups. There has been little research about the role of male primary carers in African-Caribbean and South Asian ethnicity communities despite the fact that approximately 40% of primary carers are male and predominantly spouses [22], and this will be a focus for further exploration in the study.

Lastly, people who meet the criteria for a diagnosis of dementia but have no formal diagnosis and have not accessed specialist services are largely unrepresented in research. We know that at least one third of those living with dementia in the UK have no formal diagnosis [23]. In the IDEAL-2 study, we aim to understand more about the experiences of people living with undiagnosed dementia and their primary carers and to identify factors that predict the likelihood of not receiving a diagnosis.

In summary, using both quantitative and qualitative methods, we aim to assess the key indicators of, and factors associated with, living well over the course of dementia and how these differ for particular subgroups. This wealth of evidence will also tell us which combinations of services and support are most beneficial and cost-effective, and it will offer a means to identify people at risk of decline in living well who would benefit from targeted support. The central element of IDEAL-2 will be three further yearly follow-ups of the IDEAL cohort [4]. At the time of the first follow-up we will enrich the cohort with additional participants from groups that were underrepresented in IDEAL or where small numbers were included; data from these participants will be added to the data from IDEAL waves 1–3 to allow for more extensive sub-group analyses. Continuation of the cohort will be supplemented by five linked studies; IDEAL-2 consists of six studies in total, which are described below.

### Research questions/objectives

We will address the following broad research questions aimed at creating innovations in understanding, methods, policy and practice:

1. How do key indicators of living well change as dementia progresses and what are the factors influencing these trajectories of capability to live well with dementia?
2. How do patterns of service use and costs relate to living well, and which combinations of services and support from families are both beneficial and cost-effective?
3. How can we ensure that evaluation of key indicators of living well accurately and comprehensively reflects the individual and collective experience of people living with dementia?
4. What are the best ways to include the perspectives of people with advanced dementia?
5. What can we learn about the perspectives of people with dementia from BAME groups?
6. What can we learn about the perspectives of people living with undiagnosed dementia?

## Methods/design

**Fig. 1:**
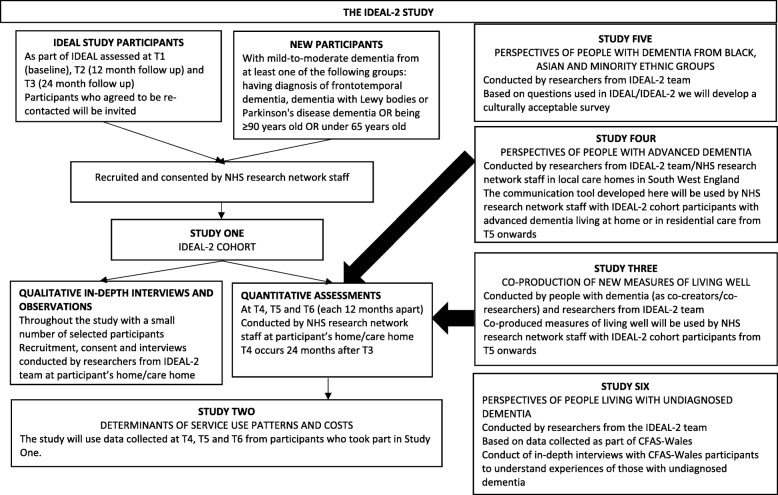
Structure of the IDEAL-2 study

The IDEAL-2 study will build on the IDEAL cohort [4] of 1547 people with mild-to-moderate dementia and 1283 primary carers. Briefly, IDEAL is a mixed-methods longitudinal study that aimed to identify what helps, or makes it difficult, to live well in the context of having dementia or caring for a person with dementia. The IDEAL cohort was recruited between July 2014 and July 2016 and assessed at three time points: T1 (baseline), T2 (12 month follow up) and T3 (24 month follow up). The central element of IDEAL-2 is three further yearly follow-ups (T4, T5 and T6) of the IDEAL cohort with T4 being 24 months after T3. At the time of the first IDEAL-2 follow-up (T4) we will enrich the cohort with additional participants with specific characteristics; their information will augment the data from IDEAL T1 and subsequent assessments to increase numbers in small sub-groups. The cohort study will be supplemented by linked studies focusing specifically on co-production of new measures of living well; on people living in residential care with advanced dementia; on people with dementia from BAME groups; and on people living with undiagnosed dementia. The IDEAL-2 programme consists of six studies in total (Fig. 1). The funding for the study started on 1st January 2018 and the study end date is 31 December 2022. The recruitment started on 25th August 2018.

### Study one: IDEAL-2 cohort: Study design

#### Primary research questions/objectives

1. How do key indicators of living well change as dementia progresses and what are the factors influencing these trajectories of capability to live well with dementia?

#### Secondary research questions/objectives

1. How do key indicators of living well differ for particular subgroups (e.g. different diagnostic groups, age-groups, gender, etc.)?
2. How is the situation of the person with dementia associated with the primary carer living well and vice versa?
3. How can we accurately identify people who are at particular risk of decline in living well?

**Fig. 2:**
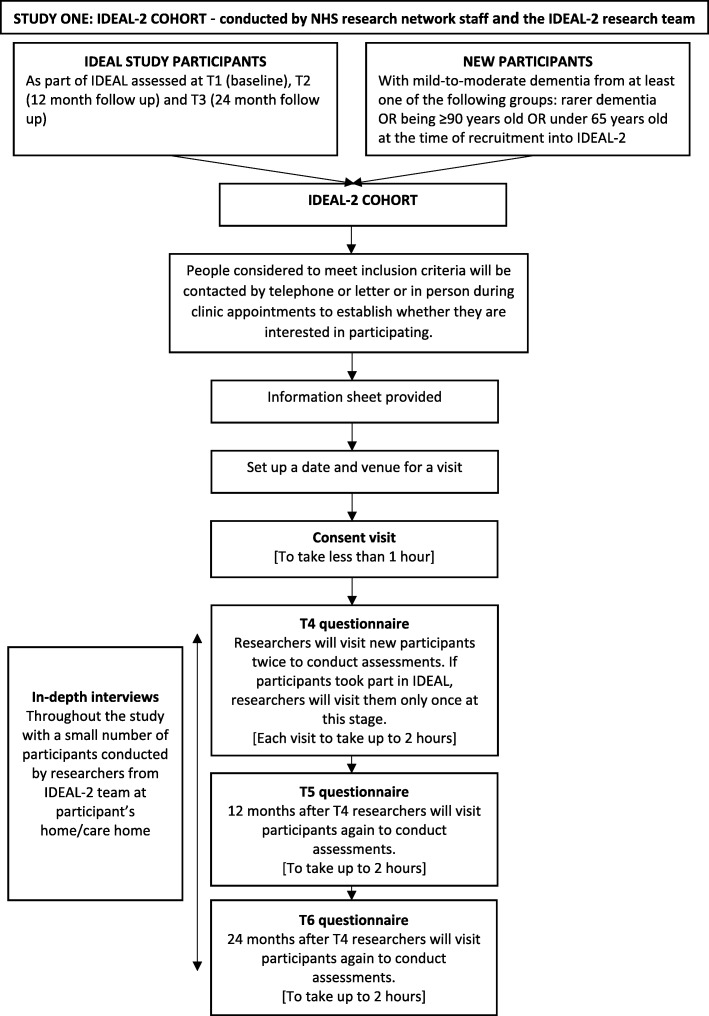
Flow of participants in the IDEAL-2 cohort

Study One is a mixed-method longitudinal study. The design and flow of participants in Study One are shown in Fig. 2. We will follow the IDEAL participants [4] at three further time-points, 12 months apart (T4, T5, and T6). In addition, we will enrich the IDEAL cohort by recruiting additional participants with mild-to-moderate dementia (a Mini-Mental State Examination (MMSE) [24] score ≥ 15) and their primary carers (where available and willing) in groups underrepresented (less than 10%) in the IDEAL cohort (people diagnosed with rarer types of dementia (frontotemporal dementia, dementia with Lewy bodies, Parkinson’s disease dementia), or ≥ 90 years old at the time of recruitment to the IDEAL-2 study). We will also recruit more participants aged under 65 (9% of the IDEAL cohort) at the request of our Patient and Public Involvement (PPI) group (Action on Living Well: Asking You - ALWAYs).

Quantitative assessments of the IDEAL-2 cohort participants using questionnaires and brief tests of cognitive ability will be conducted at T4, T5, and T6; participants and their primary carers will be assessed in their homes or usual place of residence.

A small number (*N* = 25–30) of participants experiencing key events (e.g. recent retirement or bereavement) and care transitions (e.g. moving to a care home or nursing facility) will be invited, at different time points through the IDEAL-2 cohort study, to take part in in-depth interviews. Firstly, through clinical teams, we will identify people moving into residential care to interview. We will also interview a relative and a member of care staff, and observe the person’s daily life to capture embodied aspects of living well. In addition, based on the data collected as part of the questionnaires, we will identify and interview those living with dementia who are still in employment, or who have recently retired to explore how work, and retiring from work, affects people’s experience of living with dementia. Finally, some participating primary carers will experience the death of the person with dementia during the study. We will identify these bereaved carers throughout the time of the study to explore their perceptions of end of life care and their subsequent experiences.

### Study one: IDEAL-2 cohort: Participant eligibility

Individuals were potentially eligible to participate in the IDEAL study [4] if they had a clinical diagnosis of dementia (any sub-type) and MMSE [24] score of 15 or above and lived in their own homes at the time of recruitment, with no restriction on age. IDEAL study participants will be invited to take part in the IDEAL-2 study if a) they did not exit the IDEAL study due to withdrawal or loss to follow up; and b) have previously agreed to being contacted should resources become available to find out how well they are doing after a longer period. People who lack capacity to consent will still be eligible to take part. Participants will be excluded if there is any known potential for home visits to pose a significant risk to NHS research network staff or members of IDEAL-2 study team.

New participants enriching the IDEAL cohort will be invited to take part in the IDEAL-2 study if they a) have a clinical diagnosis of dementia; b) have an MMSE score ≥ 15; c) have a good understanding of the English language to allow completion of the assessment measures; d) fit one of the criteria for additional recruitment as described earlier; and e) live in the community on entry to the study. Participants will be excluded if they a) have co-morbid terminal illness at T4; b) are unable to provide informed consent at T4; and c) represent any known potential for home visits to pose a significant risk to researchers collecting data (the same exclusion criteria as for IDEAL study entry).

Overall, participating in the IDEAL-2 cohort will not preclude participation in intervention trials or other observational studies; such participation will be noted.

In IDEAL, together with each participant with dementia, we sought to recruit a primary carer where there is one available. The same procedure will be followed for new recruitment in IDEAL-2. For the purposes of the study, we consider a primary carer to be someone who looks after the person with dementia on a regular basis, at least 3 days a week, and knows the person with dementia well enough to provide meaningful answers about his/her capability to live well. If primary carers took part in the IDEAL study, they will be invited to take part in the IDEAL-2 study even if the person with dementia whom they support does not take part in IDEAL-2. In the case of newly-recruited participants joining the IDEAL cohort, the primary carer will only be invited to take part in the study if the person with dementia consents to take part.

In cases where participants have moved into a care home, we will also recruit a member of care staff who is involved in the participant’s care and is able to provide information on the participant’s well-being.

### Study one: IDEAL-2 cohort: Setting

As the IDEAL study [4] participants were recruited from 29 NHS sites in England, Scotland and Wales, our IDEAL-2 cohort participants will be located similarly in the community across England, Scotland and Wales.

Those IDEAL participants who moved into residential care between IDEAL and IDEAL-2 will continue to be included along with a primary carer (friend/family) and a member of care staff, if available. The same will apply for all IDEAL-2 cohort participants who move into residential care at T5 or T6.

### Study one: IDEAL-2 cohort: Recruitment

New IDEAL-2 cohort participants will be recruited from memory services, old age mental health services, movement disorder services, specialist memory clinics that have a particular focus on fronto-temporal dementia, working age dementia services for people with young onset dementia and other specialist clinics within participating NHS sites in England, Scotland and Wales. Recruiters will draw on contacts with community mental health teams, general practitioner (GP) practices, social services and voluntary sector groups as appropriate. In addition to the original IDEAL NHS sites we will recruit from new NHS sites to support recruitment where necessary. NHS sites may also potentially recruit through the online Join Dementia Research register [25]. It is possible that some of the new participants will be recruited through our project partners (e.g. Alzheimer’s Society) and other agencies through promotion of the IDEAL-2 study in relevant magazines, newsletters, and through media and social media channels.

Potential participants (either from the IDEAL cohort or newly-identified) will be contacted by telephone or in person (e.g. during clinic appointments, in care homes) or by invitations approved by the Research Ethics Committee (REC) to establish whether they are interested in participating in the IDEAL-2 study. After 14 days, non-responses to the initial contact through letter or phone will be followed up by NHS research network staff once, to cover the possibility that letters and messages could be mislaid due to memory difficulties. Those participants approached with an invitation letter or face to face will receive, at the same time, a short information sheet. Those approached by telephone will be sent the same short information sheet following the call. Those participants who express interest in taking part in IDEAL-2 will be followed up by a telephone call to talk through the information sheet, and ask any questions they may have. Once participants have agreed in principle to taking part in the IDEAL-2 study, the research network staff member and participant will agree a date and a venue for the visit to take informed consent (for Study One and data linkage), and the research network staff member will send the participant the full-length information sheet and data linkage information sheet. Participants who move out of NHS site catchments areas during the course of the study will be followed up by an IDEAL-2 team member.

In the case of participants who reside in a care home, a member of care staff who is involved in the participant’s care and is able to provide information on the person’s well-being will also be invited to take part in the IDEAL-2 study either face-to-face or via phone or invitation letter. Members of care staff will be provided with an information sheet to enable them to make an informed decision about whether they would like to take part in the IDEAL-2 study. If they agree, they will be contacted to schedule a date and venue for taking informed consent and completing the questionnaires.

At T5 and T6 participants will be re-contacted by telephone or by REC-approved invitation letter or in person (e.g. during clinic appointments, in care homes) and invited to take part in the follow ups.

The steps described above will be conducted by NHS research network staff or by an IDEAL-2 team member based at the University of Exeter co-ordinating centre, all of whom will be trained in informed consent procedures as part of their Good Clinical Practice training and be familiar with General Data Protection Regulation.

Invitations to take part in the qualitative interviews together with information sheets will be sent to a purposive sample selected from participants who agreed to be approached about a further interview. The exact number of the interviewees will depend on access to the relevant subgroups and on data saturation. We will focus initially on three groups: those moving into residential care (person with dementia, family member/friend and staff member), those who are working or who have recently retired (person with dementia and family member/friend) and primary carers who are recently bereaved. After a period of a week, the invitation letters will be followed up by a telephone call to participants, to talk through the information sheet and to give them the opportunity to ask any questions they may have. Once participants have agreed in principle to take part in an interview, the researcher and participant will agree a date and a venue for the interview to take place. The interview will be preceded by further discussion and explanation of the study and the taking of written informed consent.

### Study one: IDEAL-2 cohort: Consent

It will be the responsibility of the NHS research network staff member (or an IDEAL-2 team member) to obtain written informed consent from each participant where possible prior to participation in the study, following adequate explanation of the study. Consent will be taken from the person with dementia and from a family member/friend, and for those in care homes, also from a member of care staff if available. No study procedures will be conducted prior to the participant giving consent by signing the appropriate consent form. If people with dementia lack capacity on joining IDEAL-2 but gave appropriate consent during IDEAL, no study procedures will be conducted prior to discussions with the participant’s consultee (in England and Wales) or nearest relative, guardian or welfare attorney (in Scotland).

All potential participants providing consent for entry to the IDEAL-2 cohort study will be asked about their willingness to be approached for an in-depth interview. Those participants who agree to be approached for qualitative interviews will be screened using the data they have already provided as part of the IDEAL-2 study, and those identified through purposive sampling as eligible for participation in qualitative in-depth interviews will be provided with a separate information sheet and consent form that relates specifically to their involvement in this part of the study.

This study will involve participants who may lack capacity to consent to take part in the study. NHS research network staff members (or IDEAL-2 team members) will assess whether participants have capacity to give consent to participation at T4, and to continued participation at T5 and T6. They will already have experience of interacting with and assessing people with dementia, and they will receive specific training from the research team. They will use a checklist to ensure that they make a thorough evaluation. As the IDEAL-2 cohort includes participants from England, Wales and Scotland we will follow the guidelines stipulated in the Mental Capacity Act (2005) [26] and the Adults with Incapacity (Scotland) Act 2000 [27] in relation to including participants lacking capacity in research. If the participant is judged to be unable to give informed consent at T4, different procedures will apply for IDEAL participants and new participants enriching the IDEAL cohort. Some returning IDEAL participants may now lack capacity to consent and in IDEAL we addressed this prospectively to facilitate continued inclusion in the future – participants at the time of joining the IDEAL study gave consent to be contacted should resources become available to find out how well they are doing after a longer period and they were asked to nominate a personal consultee (when participants lived in England or Wales) or legal representative such as guardian or welfare attorney or nearest relative (when participants lived in Scotland) in case they lost the capacity to consent. If a personal consultee was not available we would consult a nominated consultee (England and Wales). Therefore, if an IDEAL participant in England and Wales lacks capacity to give informed consent to join the IDEAL-2 study, we will seek the opinion of the participant’s personal consultee on the views and feelings of the participant. If an IDEAL participant in Scotland lacks capacity to give informed consent to join the IDEAL-2 study, we will ask the participant’s guardian or welfare attorney authorised to take decisions about the research or the participant’s nearest relative to give their consent. New participants with mild-to-moderate dementia recruited to the IDEAL-2 cohort must have capacity to give informed consent at the time of joining the IDEAL-2 study (T4) but again it is possible that they may lose capacity to consent through the study (at T5 and/or T6). On entry to the IDEAL-2 cohort, the issue of continuing participation will be discussed, and each participant will be asked to nominate a personal consultee (or guardian or welfare attorney or nearest relative) who can advise on the appropriateness of continued participation in the event of loss of capacity.

Ethical approval was received from the Wales Research Ethics Committee 5 – Bangor (18/WA/0111) on 20 March 2018 to conduct Study One in England and Wales and the approval from Health Research Authority was granted on 26th March 2018. Ethical approval was received from Scotland A REC (18/SS/0037) on 30th May 2018 to conduct Study One in Scotland. The study has been adopted by the National Institute of Health Research Portfolio registration number: 37955.

### Study one: IDEAL-2 cohort: Assessments

For participants who took part in IDEAL, the T4, T5 and T6 assessments will be conducted during one home visit at each time point. New participants joining the IDEAL cohort at T4 will receive an additional visit at T4 to gather baseline information comparable to that obtained at IDEAL T1. Visits to people with dementia and their primary carers are expected to last up to 2 h each. The participants will be offered a small shopping voucher (£10) as a token of appreciation for taking part in the study upon completion of the assessment at each T4, T5 and T6 assessments.

IDEAL-2 cohort participants, and their primary carers where they are contributing, will be assessed using questionnaires at each time-point by a member of NHS research network, or in a few cases by a member of the IDEAL-2 team based at the coordinating centre. NHS research network staff will receive yearly training from the study team before each assessment (at T4, T5 and T6, respectively). In the case of people with dementia, all questions will be read aloud by researchers collecting data. Primary carers will complete the questionnaires themselves.

The IDEAL-2 cohort assessment is based on our holistic framework [4]. We also took into account feedback from IDEAL study participants as well as researchers collecting data in IDEAL. The format is sufficiently flexible to allow length and level of detail to be tailored to the needs of the individual during the assessment, for example by highlighting key questions and by using show cards [28, 29]. Details of the measures that we plan to collect in the IDEAL-2 study at T4 are provided in Additional file 1.

### Study one: IDEAL-2 cohort: Withdrawal

As part of the information sheets, we will inform participants that they can withdraw at any time during the study, without having to give a reason. If a participant who lacks mental capacity to consent gives any indication of not wanting to continue to take part, we will withdraw the participant from the study. The participant will also be withdrawn if the personal/nominated consultee (or guardian or welfare attorney or nearest relative) advises against continued participation.

If participants withdraw, we will use the information participants provide up to that point in line with the General Data Protection Regulation, unless they indicate that they do not want us to.

### Study one: IDEAL-2 cohort: Adverse events

This is a low-risk observational study and, accordingly, no formal adverse event monitoring is planned. However, events may come to the attention of the research team which will be recorded using an adverse event reporting form. Research staff working on the IDEAL-2 study will notify the local Principal Investigator or Chief Investigator when such events occur.

### Study one: IDEAL-2 cohort: Sample size calculation

For the IDEAL-2 cohort, the participants will be re-consented from the ongoing IDEAL study. Between 2014 and 2016 IDEAL recruited 1547 people with dementia; 83% had a primary carer participating giving a total of 1283 primary carers. For the present study, the sample size will be determined by the number of IDEAL participants who agree to take part in the IDEAL-2 study. In addition we will recruit new participants, and their primary carers where available and willing, to enrich the IDEAL cohort in specific sub-groups that constituted fewer than 10% of the cohort at T1. These will be people diagnosed with rarer types of dementia (frontotemporal dementia, dementia with Lewy bodies, Parkinson’s disease dementia), and/or aged either < 60 or ≥ 90 years at the time of recruitment. Combining data from these new participants with data collected from IDEAL participants at T1 will improve our capability to conduct meaningful sub-group analyses. Once we reach the target for each enrichment group (increasing the number in that particular sub-group to the equivalent of 10% of the IDEAL cohort at T1) we will stop the recruitment of additional participants.

### Study one: IDEAL-2 cohort: Analysis plan

The IDEAL-2 cohort will provide longitudinal data on living well measures as well as social, psychological and physical health factors in people with dementia and their primary carers. Longitudinal analysis will investigate changes in key variables over time, examine predictors of capability to live well or decline in capability to live well, and compare different patterns across subgroups. As well as analysing data for people with dementia and carers separately, we will focus on the dyadic relationships between people with dementia and their primary carers and examine how the situation of each partner influences the well-being of the other over time using the actor-partner independence model, extended to also incorporate a third level of data where the carer acts as an informant on behalf of the person with dementia.

The qualitative interview data collected as part of the IDEAL-2 cohort and the open-ended questions collected using questionnaires will be analysed using a thematic approach [30].

### Study two: Determinants of service use patterns and costs: Study design

#### Primary research questions/objectives

1. How do patterns of service use and costs relate to living well, and which combinations of services and support from families are both beneficial and cost-effective?

#### Secondary research questions/objectives

1. What are the services that people with dementia use, what influences service use and what are the associated costs?
2. How do services and unpaid care influence the capability to live well with dementia?

This study involves analysis of IDEAL-2 cohort data collected under Study One from a health economic perspective. Therefore, as part of the IDEAL-2 assessments we will collect information on service use and the associated costs. We will describe the information using standard descriptive statistical methods, multilevel and linear growth curve modelling. Service use and costs will be summarised by key characteristics of sample members with dementia (including age, gender, type of dementia, severity of needs, comorbidities) and locality. Trends over time (i.e. from one follow up to another) will also be reported. To calculate costs (by service, sector or overall), the service use measures in IDEAL and IDEAL-2 will be weighted by their unit costs and aggregated. Unit costs will reflect long-run marginal opportunity costs and will be drawn from publicly available sources wherever possible, such as the annual Personal Social Services Research Unit volume [31] and NHS Reference Costs [32]. Costs will also be attached to unpaid care, estimated from information on volume and type of support, the opportunity cost of lost work (wage rate) for primary carers in paid employment, and replacement cost for those not in paid employment based on (for example) the cost of a home care worker. Medication costs will be included, based on current prices to the NHS.

We will then explore how service use patterns and their costs are associated with individuals’ needs and a range of other personal characteristics and contextual factors, adjusting for all other relevant factors. These analyses, including estimation of cost functions [33], will be based on multilevel, general linear and structural equation modelling, paying attention to possible non-normal distributions, non-linear associations and endogeneity. Previous research in other fields demonstrates the feasibility and usefulness of such approaches [34–36]. For instance, in the analyses we will examine whether the levels and composition of costs differ between groups with different characteristics (e.g. particular types of dementia or gender), whether the rate of change of these costs varies between these groups and whether that rate is linear or non-linear.

A parallel set of analyses will examine the influences of services and unpaid care on quality of life and other outcomes, again after adjusting for all other relevant factors. These further multivariate analyses (‘production functions’ [37]) have not been explored previously in the dementia field; they will be valuable in their own right, helping to inform policy and practice discussions about service deployment, cost and funding, but they can also be used in what are effectively ‘naturalistic’ cost-effectiveness analyses of different types and combinations of support for people with dementia and their primary carers, conducted in non-experimental, real-life conditions. These analyses will again include general linear and structural equation modelling, taking into account the possibility of non-normal distributions, non-linear associations and endogeneity.

To examine cost-effectiveness, we will include indicators of interventions in the multivariate analyses of costs and outcomes, using estimated parameters from regression models to create incremental cost-effectiveness ratios (and confidence intervals around them). Again, we can build on previously developed methods by ourselves and others [38–40]. By ‘interventions’ we mean the services (individual or in combination) used in the naturalistic context of a cohort design such as the IDEAL-2 study.

We plan to achieve comprehensive linkage to routine health service datasets in the IDEAL-2 study, which will allow us to compare responses from sample members and administrative sources, as well as provide a contingency in the event of missing data from the IDEAL-2 study questionnaires, providing a comprehensive picture of service use.

### Study two: Determinants of service use patterns and costs: Consent

This study involves analysis of IDEAL-2 cohort data collected under Study One from a health economic perspective. Consent will be therefore sought for this study as part of Study One: IDEAL-2 cohort. In addition, our analyses will be enhanced, subject to participant consent, by data linkage to routine data. We will obtain separate consent in written form from participants with dementia to data linkage at T4. If an IDEAL participant in England and Wales lacks capacity to give informed consent to data linkage as part of IDEAL-2, we will seek the opinion of the participant’s personal consultee on the views and feelings of the participant. If an IDEAL participant in Scotland lacks capacity to give informed consent to data linkage, we will ask the participant’s guardian or welfare attorney authorised to take decisions about the research or the participant’s nearest relative to give their consent.

Ethical approval was received from the Wales Research Ethics Committee 5 – Bangor (18/WA/0111) on 20 March 2018 to conduct Study Two in England and Wales and the approval from Health Research Authority was granted on 26th March 2018. Ethical approval was received from Scotland A REC (18/SS/0037) on 30th May 2018 to conduct Study Two in Scotland.

### Study three: Co-production of new measures of living well: Study design

#### Primary research questions/objectives

1. How can we ensure that evaluation of key indicators of living well accurately and comprehensively reflects the individual and collective experience of people living with dementia?

#### Secondary research questions/objectives

1. How can we develop a standardised and personalised measure of living well that reflect the experience of people with dementia?
2. How can these living well measures be made accessible for people with dementia?

As part of the IDEAL-2 study we will co-produce two measures to aid the evaluation of the capability to live well with dementia: a standardised questionnaire and an individualised measure based on ‘personal questionnaire’ methodology. The standardised questionnaire will be a traditional type of measure where all participants are given the same statements to rate on a Likert-type scale. In the personal questionnaire approach, participants will generate their own statements to rate on a similar Likert-type scale; this is particularly useful for identifying changes over time, and is used for example in psychotherapy research [41].

The co-production process will include four phases: preparation; development; piloting; and testing. It will be facilitated by our partner organisation Innovations in Dementia [42]. In Study Three, people with dementia will be invited to act as co-researchers and will work in equal partnership with the researchers from the IDEAL-2 team. Additional people with dementia will be recruited as participants for the piloting and testing phases.

In the preparation phase, as a first step, researchers from the IDEAL-2 team will review the data that were collected through in-depth face to face interviews as part of the IDEAL study [4] and identify categories and themes relevant to perceptions of quality of life, well-being and satisfaction with life. As a next step, the co-production team made up of people with dementia and IDEAL-2 researchers will be invited to review this information and suggest any additional topics not covered in the first step that they think are important. The aim of the preparation phase is to produce a list of topics and themes that are representative of the views on living well by people with dementia. These will serve as a starting point for the development of the co-produced measures.

In the development phase, the co-production team will review existing measures of quality of life, well-being and satisfaction with life e.g. the use of language, response options, and mode of administration, and consider their strengths and limitations. Based on the preferences of co-researchers, the team will refine the themes from the preparation phase and develop specific items and response options for the co-produced questionnaire.

We aim to include 8–10 people with dementia to be co-researchers. There are no inclusion or exclusion criteria for co-researchers, other than that they have to be a person living with dementia who would like to be involved in the research. We will recruit these co-researchers from the Dementia Engagement and Empowerment Project (DEEP) groups (through Innovations in Dementia [42]) in North West England. We hope to have a diverse mix of people participating, including people from a range of ethnic backgrounds and at varying stages of dementia. The nature of the co-researcher role will be made clear to people before they are offered the opportunity, so that they are able to decide if the role is right for them.

In the piloting phase, both the standardised measure and the personalised measure of living well will be tested with people with dementia (participants). Participants will complete both measures with a member of the co-production team. The co-production team will use the feedback from the piloting phase to make final adjustments to both measures, and will also produce guidelines for administration.

Lastly, in the testing phase, we will test both of the co-produced measures with people with dementia (participants) twice, with a two-week interval, to measure test-retest reliability. Researchers from the IDEAL-2 team will then review the findings from this phase and refine the measures if necessary. All members of the co-production team will have access to the final products and have the opportunity to be engaged in dissemination. Depending on the timeline, we hope to introduce both of the co-produced measures in the T5 and/or T6 assessments of the IDEAL-2 cohort.

### Study three: Co-production of new measures of living well: Consent

Co-researchers will be given sufficient information about their role and what will be expected of them during the research, with information leaflets reviewed and approved by the University of Exeter REC. As they will act as co-researchers (as opposed to participants) they will not be asked to provide written consent, as implied consent is more appropriate to co-production [43]. Innovations in Dementia [42] who will facilitate Study Three will work to their own ethical framework. During the meetings, a set of clearly visible reminders will be displayed focusing on co-researchers’ rights, e.g. voluntary participation; everything shared in the group space will be anonymised; and that they are free to stop participating in the project at any time. Participants (people with dementia) for the piloting and testing phase will be asked to provide informed consent through information sheets and the signing of consent forms, both of which will be designed with co-researchers.

Ethical approval for Study Three was received from the CLES – Psychology Ethics Committee (eCLESPsy000569 v4.0) on 22nd May 2018.

### Study four: Perspectives of people living with advanced dementia: Study design

#### Primary research questions/objectives

1. What are the best ways to include the perspectives of people with advanced dementia?

#### Secondary research questions/objectives

1. What are the best methods that enable people with advanced dementia to give their views about their own capability to live well?
2. What are the domains related to living well that people with advanced dementia consider to be most relevant and important?
3. How can we improve the lives of people with advanced dementia?

In this study, we will develop and test a communication toolkit which will be used to support the inclusion of the perspectives of people with advanced dementia who still use at least some verbal communication in research. We aim to adopt a comprehensive approach to understanding the ways in which people with advanced dementia communicate their thoughts, feelings and preferences, and how we can use this knowledge to promote inclusion of this particularly vulnerable group in research.

Currently, several methods exist that may facilitate communication with people with advanced dementia such as picture cards [11], talking mats [44–46], brief observational methods such as AwareCare [47] and the Positive Response Schedule [48], and a range of other examples [49–54]. Namaste care also focuses on non-verbal communication [55, 56]. To inform the development of a communication toolkit to promote inclusion in research we will conduct a systematic review of these methods. We will interview dementia professionals (e.g. speech and language therapists, occupational therapists, psychologists), and other experts in non-verbal communication to identify best practice in communicating with people living with advanced dementia. In addition, we will conduct focus groups, observations and workshop sessions.

We will use what we have learned to develop a communication toolkit to train and support researchers in gaining the perspective of people with advanced dementia who still have some capability for verbal communication. We will then field-test and refine the toolkit in an iterative process of engagement with people who have advanced dementia living in care homes, and test its robustness with a further group of care home residents with advanced dementia, who will be followed up after 12 months.

There will be a degree of purposive sampling, to make sure that we have a mixture of people with advanced dementia (different ages, different homes, different ethnicities if possible, near-to-equal gender split, different sub-types of dementia, and different stages of ‘advanced’ dementia). People will be potentially eligible to take part in Study Four if they meet the following inclusion criteria: a) they have a formal clinical diagnosis of any sub-type of dementia; and b) they have moderate to severe dementia as determined by any suitable assessment method. Participants will be excluded if they are a) acutely ill; b) thought to be imminently dying; or c) too frail to engage with research staff.

Participants with advanced dementia will be recruited primarily from a range of care homes with which the research team has prior links. Potential participants will in most cases be identified and recruited by care home staff following discussion of inclusion and exclusion criteria with a member of the research team. We will ask staff to approach potentially eligible participants to explain the study and provide them with a participant information sheet in order to aid the invitation process. Once it is ascertained that the person with dementia is happy to be approached by the researcher, the researcher will then talk to the potential participant. The researcher will introduce him- or herself and re-explain the research in a simplified and clear manner. If potential participants are distressed or object to the introduction, they will be thanked for their time and not included in the research.

Once the toolkit has been fully field-tested, we aim to train researchers collecting data in the IDEAL-2 cohort to use the toolkit to support their engagement with participants with advanced dementia living at home or in residential care at T5 and T6, and to explore how effective this is in enabling us to continue to include participants in the IDEAL-2 assessments whose dementia is now at an advanced stage.

### Study four: Perspectives of people living with advanced dementia: Consent

It will be the responsibility of the NHS research network staff member (or an IDEAL-2 team member) to obtain written informed consent from each participant where possible prior to participation in the study, following adequate explanation of the study. We will be informed by DEEP and Alzheimer’s Society guidelines alongside input from the ALWAYs group about using clear, transparent and user-friendly information sheets and consent forms. We will also work with care home staff to understand the most appropriate way to engage with the individual during the information process. As participants with advanced dementia will all be living in England, the research team will follow the guidelines of the Mental Capacity Act (2005) [26] and presume the potential participant has the mental capacity to consent prior to a formal mental capacity assessment. Residents who have capacity will provide formal, written consent. Study procedures will only be conducted where the participant has given consent by signing the consent form or a personal or nominated consultee has deemed it appropriate for the person who lacks capacity to participate. Where it is established (using the criteria set out in the Mental Capacity Act 2005 [26]), that a person lacks the capacity to consent, then an approach will be made to someone close to the resident who is able to act as a personal consultee. If such a person is not identifiable, a nominated consultee (e.g. GP or health professional) will be sought (in accordance with section 32 [3] of the Mental Capacity Act 2005 [26]). Consultees will be asked for advice as to whether (a) the person should take part in the project, and (b) what the person’s wishes and feelings about taking part in the project would be likely to be if the person had capacity to make the decision for him- or herself (Mental Capacity Act 2005 [26], section 32 [4]). If the consultee’s advice is that participation in the research can go ahead, the researcher will proceed but will be sensitive to the responses of the person with dementia. If by any means it is thought that the person with dementia is signalling that he or she does not wish to participate (or wishes to stop participating) – by words, gestures, attitude, other vocalisations, emotional displays, etc. – then the person will not be included in the study (or his or her participation will cease). The researcher will check continually that the person is still consenting or assenting. Written prompts will be used to facilitate this and to re-explain what is happening and that the person is free to say whether he or she wishes to stop.

Ethical approval was received from the Wales Research Ethics Committee 5 – Bangor (18/WA/0111) on 20 March 2018 to conduct Study Four in England and Wales and the approval from Health Research Authority was granted on 26th March 2018.

### Study five: Perspectives of people from BAME groups: Study design

#### Primary research questions/objectives

1. What can we learn about the perspectives of people with dementia from BAME groups?

#### Secondary research questions/objectives

1. Which factors support living well for people with dementia and primary carers from BAME communities?
2. How can we best support the inclusion of people with dementia from BAME groups in future research and health care service?

In this study, we will explore the potential for including the perspectives of people with dementia from BAME groups by focussing on people of African-Caribbean and South Asian ethnicity. In Phase One we will work with community groups to build relationships and trust, consulting community leaders and gatekeepers and offering information and awareness sessions to develop a strategy for identifying people with dementia. In Phase Two we will conduct qualitative interviews with people with dementia and their primary carers from each community, to explore their experiences and examine the feasibility of a wider survey. In Phase Three we will work with people from these communities to develop and implement a questionnaire based on the IDEAL-2 cohort T4 quantitative assessment for newly-recruited participants that is perceived as culturally-acceptable by African-Caribbean and South Asian communities. Once the questionnaire is developed, we will recruit and assess a larger group of people with dementia from each community, with a primary carer where available, and follow them up 12 months later. In addition, we will develop a more in-depth approach to investigating the experiences and needs of male primary carers of African-Caribbean and South Asian ethnicity.

### Study five: Perspectives of people from BAME groups: Consent

Written consent will be sought from all participants taking part in different stages of this study (Phase 1–3).

Ethical approval for Study Five was received from College of Health and Life Sciences Research Ethics Committee, Brunel University London (10598-LR-Mar/2018–12350-2) on 19th April 2018 for the community leader interviews.

### Study six: Perspectives of people living with undiagnosed dementia: Study design

#### Primary research questions/objectives

1. What can we learn about the perspectives of people living with undiagnosed dementia?

#### Secondary research questions/objectives

1. What is the evidence on the experiences of people living with undiagnosed dementia and their primary carers?
2. What is the evidence on how people living with undiagnosed dementia differ from their diagnosed counterparts?
3. What factors predict the likelihood of not receiving a diagnosis of dementia?

To learn more about the experiences of people living with undiagnosed dementia, we will use the Cognitive Function and Ageing Study (CFAS)-Wales cohort [57] to identify participants living with undiagnosed dementia and examine their experience. Using the CFAS-Wales data we aim to identify factors that predict the likelihood of not receiving a dementia diagnosis. In addition, we will recruit and interview participants taking part in the CFAS-Wales study [57] whose data indicated that they met criteria for dementia, determined by a diagnostic algorithm and clinician review, but were undiagnosed as indicated by linkage of study data with GP registers. In each case we will also interview a family member where available. These interviews will provide insight into the role that diagnosis plays (or does not play) in the everyday lives of people living with or affected by dementia.

### Study six: Perspectives of people living with undiagnosed dementia: Consent

Written consent will be sought from all participants who will take part in qualitative interviews.

Ethical approval for Study Six was received from the Wales Research Ethics Committee 5 Bangor (10/WNo01/37) on 3rd July 2018 as an amendment to the CFAS-Wales study ethics application (reference number 10/WNo01/37; Chief Investigator: Professor Robert Woods).

## Discussion

Large longitudinal studies are rare in social science research focusing on people with dementia, and little is known about how capability to live well changes as dementia progresses [16]. IDEAL-2 offers the opportunity to address this evidence gap with data of exceptional scope and duration. Together, the IDEAL and IDEAL-2 studies will constitute the largest study of living well with dementia in Great Britain and possibly worldwide. This will provide us with much-needed evidence about the key indicators of, and factors associated with, living well over the course of dementia and how these differ for particular subgroups. It will tell us which combinations of services and support are most beneficial and cost-effective. At the individual level it will provide tools with which to identify people at risk of decline in well-being who would benefit from targeted support.

The evidence IDEAL-2 provides will benefit the lives of people with dementia and their primary carers in the future by shaping developments in policy, practice, research and service provision. More broadly, it will contribute to improving quality of life by enhancing care practices, shaping community initiatives and widening public understanding of how to support people with dementia. It will provide a measure of living well co-developed by people living with dementia. It will lead to recommendations for a sustainable and accessible approach to monitoring ability to live well with dementia and identifying unmet needs, with the potential to be scaled up to population level. The availability of this rich resource will also make it possible to contrast the experience of our cohort with that of their counterparts without dementia through comparisons with data from existing longitudinal studies. IDEAL-2 will contribute to building capacity in dementia research and enhancing workforce skills, with a strong focus on intervention development that will catalyse longer-term improvements in care and support, and will create a unique data set that other researchers can use. Finally, ALWAYs group members have told us that taking part in a study of this kind can offer individual participants a valuable opportunity for reflection and can help in coming to terms with the changes they are experiencing.

## Additional file

Additional file 1: Content of the IDEAL-2 study questionnaires administered to the cohort at T4 (DOCX 115 kb)

## Abbreviations

ALWAYs: Action on Living Well: Asking You (Patient and Public Involvement group)
BAME: Black, Asian and minority ethnic groups
CFAS: Cognitive Function and Ageing Study
DEEP: Dementia Engagement and Empowerment Project network
GP(s): General Practitioner(s)
MMSE: Mini-Mental State Examination
NHS: National Health Service
PPI: Patient and Public Involvement
REC: Research Ethics Committee
IDEAL-2: The IDEAL-2 study: Improving the experience of Dementia and Enhancing Active Life: a longitudinal perspective on living well with dementia
UK: United Kingdom

## References

[1] Cooper C, Mukadam N, Katona C, Lyketsos CG, Ames D, Rabins P (2012). Systematic review of the effectiveness of non-pharmacological interventions to improve quality of life of people with dementia. Int Psychogeriatr.

[2] Cooper C, Mukadam N, Katona C, Lyketsos CG, Blazer D, Ames D (2013). Systematic review of the effectiveness of pharmacologic interventions to improve quality of life and well-being in people with dementia. Am J Geriatr Psychiatry.

[3] Martyr Anthony, Nelis Sharon M., Quinn Catherine, Wu Yu-Tzu, Lamont Ruth A., Henderson Catherine, Clarke Rachel, Hindle John V., Thom Jeanette M., Jones Ian Rees, Morris Robin G., Rusted Jennifer M., Victor Christina R., Clare Linda (2018). Living well with dementia: a systematic review and correlational meta-analysis of factors associated with quality of life, well-being and life satisfaction in people with dementia. Psychological Medicine.

[4] Clare L, Nelis SM, Quinn C, Martyr A, Henderson C, Hindle JV (2014). Improving the experience of dementia and enhancing active life--living well with dementia: study protocol for the IDEAL study. Health Qual Life Outcomes.

[5] Logsdon RG, Gibbons LE, McCurry SM, Teri L (1999). Quality of life in Alzheimer’s disease: patient and caregiver reports. J Ment Health Aging.

[6] Bech P (2004). Measuring the dimension of psychological general well-being by the WHO-5. Qual Life Newsletter.

[7] Diener E, Emmons RA, Larsen RJ, Griffin S (1985). The satisfaction with life scale. J Pers Assess.

[8] Bowling A, Rowe G, Adams S, Sands P, Samsi K, Crane M (2015). Quality of life in dementia: a systematically conducted narrative review of dementia-specific measurement scales. Aging Ment Health.

[9] Hughes JC (2003). Quality of life in dementia: an ethical and philosophical perspective. Expert Rev Pharmacoecon Outcomes Res.

[10] Missotten P, Dupuis G, Adam S (2016). Dementia-specific quality of life instruments: a conceptual analysis. Int Psychogeriatr.

[11] My name is not dementia: People with dementia discuss quality of life indicators: Alzheimer’s Society; 2010 [Available from: http://www.cardi.ie/userfiles/My_name_is_not_dementia_report%5B1%5D.pdf.

[12] Castro-Monteiro E, Forjaz MJ, Ayala A, Rodriguez-Blazquez C, Fernandez-Mayoralas G, Diaz-Redondo A (2014). Change and predictors of quality of life in institutionalized older adults with dementia. Qual Life Res Int J Qual Life Asp Treat Care Rehab.

[13] Cordner Z, Blass DM, Rabins PV, Black BS (2010). Quality of life in nursing home residents with advanced dementia. J Am Geriatr Soc.

[14] Mjorud M, Rosvik J, Rokstad AM, Kirkevold M, Engedal K (2014). Variables associated with change in quality of life among persons with dementia in nursing homes: a 10 months follow-up study. PLoS One.

[15] Clare L, Rowlands J, Bruce E, Surr C, Downs M (2008). The experience of living with dementia in residential care: an interpretative phenomenological analysis. The Gerontologist.

[16] Clare L, Quinn C, Hoare Z, Whitaker R, Woods RT (2014). Care staff and family member perspectives on quality of life in people with very severe dementia in long-term care: a cross-sectional study. Health Qual Life Outcomes.

[17] Round J, Sampson EL (2014). Jones L. a framework for understanding quality of life in individuals without capacity. Qual Life Res Int J Qual Life Asp Treat Care Rehab.

[18] Adelman S, Blanchard M, Rait G, Leavey G, Livingston G (2011). Prevalence of dementia in African-Caribbean compared with UK-born white older people: two-stage cross-sectional study. Br J Psychiatry J Ment Sci.

[19] Mayeda ER, Glymour MM, Quesenberry CP, Whitmer RA (2016). Inequalities in dementia incidence between six racial and ethnic groups over 14 years. Alzheimers Dement.

[20] Moriarty JSN, Robinson J (2011). SCIE Research briefing 35: black and minority ethnic people with dementia and their access to support and services.

[21] Victor CR, Burholt V, Martin W (2012). Loneliness and ethnic minority elders in Great Britain: an exploratory study. J Cross Cult Gerontol.

[22] Victor CR, Dobbs C, Burholt V. Caring in minority communities in England and Wales: SAGE Open special issue. In press.

[23] BC PE (2015). Dementia - An overview of policy and services, and statistics on prevalence. (Briefing Paper 07007).

[24] Folstein MF, Folstein SE, McHugh PR (1975). “Mini-mental state”. A practical method for grading the cognitive state of patients for the clinician. J Psychiatr Res.

[25] Join dementia research [Available from: https://www.joindementiaresearch.nihr.ac.uk/.

[26] Mental Capacity Act 2005. Available from: https://www.legislation.gov.uk/ukpga/2005/9/pdfs/ukpga_20050009_en.pdf.

[27] The Adults with Incapacity (Scotland) Act 2000. Available from: https://www.gov.scot/Publications/2008/03/25120154/1.

[28] Nind M. Conducting qualitative research with people with learning, communication and other disabilities: methodological challenges: ESRC National Centre for Research Methods Review Paper; 2008. http://eprints.ncrm.ac.uk/491/1/MethodsReviewPaperNCRM-012.pdf.

[29] Beadle-Brown JRS, Windle K, Holder J, Turnpenny A, Smith N, Richardson L, Whelton B (2012). Engagement of people with long - term conditions in health and social care research: barriers and facilitators to capturing the views of seldom-heard populations.

[30] Charmaz K, Holstein JGJ (2008). Constructionism and the grounded theory method. Handbook of constructionist research.

[31] Curtis L, Burns A. Unit costs of health and social care 2016, Personal Social Services Research Unit. Canterbury: University of Kent; 2016.

[32] Department of Health (2016). National Health Service Schedule of Reference Costs.

[33] Knapp M (1984). The economics of social care.

[34] Beecham J, Hallam A, Knapp M, Carpenter J, Cambridge P, Forrester-Jones R (2004). Twelve years on: service use and costs for people with mental health problems who left psychiatric hospital. J Ment Health.

[35] Evans-Lacko S, Takizawa R, Brimblecombe N, King D, Knapp M, Maughan B (2017). Childhood bullying victimization is associated with use of mental health services over five decades: a longitudinal nationally representative cohort study. Psychol Med.

[36] Knapp M, King D, Healey A, Thomas C (2011). Economic outcomes in adulthood and their associations with antisocial conduct, attention deficit and anxiety problems in childhood. J Ment Health Policy Econ.

[37] Davies B, Fernandez, J, Nomer B. Equity and efficiency policy in community care: needs, service productivities, efficiencies, and their implications. Published by Ashgate, Aldersho; 2000. p. 494. ISBN 0546 1281.

[38] Forder J, Malley J, Towers AM, Netten A (2014). Using cost-effectiveness estimates from survey data to guide commissioning: an application to home care. Health Econ.

[39] Knapp M, Windmeijer F, Brown J, Kontodimas S, Tzivelekis S, Haro JM (2008). Cost-utility analysis of treatment with olanzapine compared with other antipsychotic treatments in patients with schizophrenia in the pan-European SOHO study. PharmacoEconomics.

[40] Windmeijer F, Kontodimas S, Knapp M, Brown J, Haro JM (2006). Methodological approach for assessing the cost-effectiveness of treatments using longitudinal observational data: the SOHO study. Int J Technol Assess Health Care.

[41] Elliott R, Wagner J, Sales CM, Rodgers B, Alves P, Cafe MJ (2016). Psychometrics of the personal questionnaire: a client-generated outcome measure. Psychol Assess.

[42] Innovations in Dementia [Available from: http://www.innovationsindementia.org.uk/.

[43] Thomas-Hughes H (2018). Ethical ‘mess’ in co-produced research: reflections from a U.K.-based case study. Int J Soc Res Methodol.

[44] Murphy J, Gray CM, van Achterberg T, Wyke S, Cox S (2010). The effectiveness of the talking Mats framework in helping people with dementia to express their views on well-being. Dementia.

[45] Murphy J, Oliver T (2013). The use of talking Mats to support people with dementia and their carers to make decisions together. Health Soc Care Community.

[46] Murphy J, Tester S, Hubbard G, Downs M, MacDonald C (2005). Enabling frail older people with a communication difficulty to express their views: the use of talking Mats as an interview tool. Health Soc Care Commun.

[47] Clare L, Whitaker R, Quinn C, Jelley H, Hoare Z, Woods B (2012). AwareCare: development and validation of an observational measure of awareness in people with severe dementia. Neuropsychol Rehabil.

[48] Perrin T (1997). The positive response schedule for severe dementia. Aging Ment Health.

[49] Astell AJ, Ellis MP, Alm N, Dye R, Gowans G (2010). Stimulating people with dementia to reminisce using personal and generic photographs. J Comput Health.

[50] Astell AJ, Ellis MP, Bernardi L, Alm N, Dye R, Gowans G (2010). Using a touch screen computer to support relationships between people with dementia and caregivers. Interact Comput.

[51] Ellis M, Astell AJ (2017). Adaptive interaction and dementia: how to communicate without speech. Lonsdon and.

[52] Killick J, Allan K (2005). Good sunset project: quality of life in advanced dementia. J Dement Care.

[53] Killick J, Allan K (2006). Making contact with those close to death. J Dement Care.

[54] Killick J, Allan K (2006). The getting through initiative: inside the interactions. J Dement Care.

[55] Simard J (2013). The end - of - life Namaste care™ program for people with dementia. 2nd edition ed. Baltimore.

[56] Stacpoole M, Hockley J, Thompsell A, Simard J, Volicer L (2015). The Namaste care programme can reduce behavioural symptoms in care home residents with advanced dementia. Int J Geriatr Psychiatry.

[57] McCallum C, Rooksby J, Gray CM (2018). Evaluating the impact of physical activity apps and wearables: interdisciplinary review. JMIR mHealth uHealth.

