# Supplementary material for: Protocol for the IDEAL-2 longitudinal study: following the experiences of people with dementia and their primary carers to understand what contributes to living well with dementia and enhances active life
Source: BMC Public Health. 2018 Oct 30;18:1214. doi: 10.1186/s12889-018-6129-7 (PMC6208177; doi:10.1186/s12889-018-6129-7)
Supplement: Supplementary file 1 — Content of the IDEAL-2 study questionnaires administered to the cohort at T4 (DOCX 115 kb) [file 12889_2018_6129_MOESM1_ESM.docx]

**Additional Files**

**Additional file 1: Content of the IDEAL-2 study questionnaires administered to the cohort at T4**

**Protocol for the IDEAL-2 longitudinal study: following the experiences of people with dementia and their primary carers to understand what contributes to living well with dementia and enhances active life**

Barbora Silarova, Sharon M Nelis, Rosalie M Ashworth, Clive Ballard, Marta Bieńkiewicz, Cate Henderson, Alexandra Hillman, John V Hindle, Julian C Hughes, Ruth A Lamont, Rachael Litherland, Ian R Jones, Roy W Jones, Martin Knapp, Piers Kotting, Anthony Martyr, Fiona E Matthews, Robin G Morris, Catherine Quinn, Jemma Regan, Jennifer M Rusted, Eleanor Ann van den Heuvel, Christina R Victor, Yu-Tzu Wu, Linda Clare

The IDEAL-2 study measures are provided in Table 1-2, indicating who will complete the measures and at what stage of the study they will be administered. New participants with dementia (and their carers where available) who did not take part in the IDEAL study will be required to complete further measurements in visit 1 (v1) at T4.

**Table 1**. Questions about the person with dementia

| **SOURCE OF INFORMATION** | **Person with dementia** | | **Relative/friend** | | **Member of care staff** |
| --- | --- | --- | --- | --- | --- |
| **TIME OF DATA COLLECTION** | **T4** | | **T4** | | **T4** |
| **MEASURES** | **v1** | **v2** | **v1** | **v2** | **v2** |
| **PERSONAL CHARACTERISTICS** |  |  |  |  |  |
| Location and living situation ([1](#_ENREF_1)) |  | **●** |  | ● |  |
| Date of birth |  | **●** |  |  |  |
| Gender |  | **●** |  |  |  |
| Ethnicity | ● |  |  |  |  |
| Marital status |  | **●** |  |  |  |
| Country of birth | ● |  |  |  |  |
| Main language | ● |  |  |  |  |
| Sexual orientation | ● |  |  |  |  |
| Ownership of pets ([2](#_ENREF_2)) | ● |  |  |  |  |
| Number of children | ● |  |  |  |  |
| Religion | ● |  |  |  |  |
| Information on dementia diagnostic status |  | **●** |  |  |  |
| **SOCIAL LOCATION** |  |  |  |  |  |
| Occupation | ● | **●** |  |  |  |
| Social comparison | ● |  |  |  |  |
| The MacArthur Scale of Subjective Social Status - position on societal and community ladders ([3](#_ENREF_3)) |  | **●** |  |  |  |
| **CAPITALS, ASSETS AND RESOURCES** |  |  |  |  |  |
| *Social capital - personal relations, social network support, civic engagement, trust and co-operative norms* |  |  |  |  |  |
| Social networks and support - satisfaction ([4](#_ENREF_4), [5](#_ENREF_5)) |  | **●** |  | ● |  |
| Social networks and support – frequency of contact ([4](#_ENREF_4), [5](#_ENREF_5)) | **●** |  | **●** |  |  |
| Social participation - civic engagement, volunteering (ONS) ([6](#_ENREF_6)) |  | **●** |  |  |  |
| Reciprocity and local trust in neighbourhood (Office for National Statistic) ([6](#_ENREF_6)) |  | **●** |  |  |  |
| *Cultural capital - institutionalised (education); incorporated (cultural participation)* |  |  |  |  |  |
| Education | ● |  |  |  |  |
| *Economic capital* |  |  |  |  |  |
| Income ([7](#_ENREF_7)) |  | **●** |  |  |  |
| *Other capital* |  |  |  |  |  |
| Activity engagement ([8-10](#_ENREF_8)) |  | **●** |  | ● |  |
| **ENVIRONMENT** |  |  |  |  |  |
| Life space ([11](#_ENREF_11)) |  | ● |  |  |  |
| Neighbourhood - green and blue spaces ([12](#_ENREF_12)) |  | ● |  | ● |  |
| **PSYCHOLOGICAL CHARACTERISTICS AND PSYCHOLOGICAL HEALTH** |  |  |  |  |  |
| Personality - 5 dimensions ([13](#_ENREF_13)) | ● |  |  |  |  |
| Religious practice ([14](#_ENREF_14)) | ● |  |  |  |  |
| Spirituality ([15-17](#_ENREF_15)) | ● |  |  |  |  |
| Optimism - Life Orientation Test ([18](#_ENREF_18)) | **●** |  |  |  |  |
| Optimism - Life Orientation Test - single item ([18](#_ENREF_18)) |  | **●** |  |  |  |
| Sense of self (continuity/discontinuity) |  | **●** |  |  |  |
| Self-esteem - single item ([19](#_ENREF_19)) |  | **●** |  |  |  |
| Self-esteem - Rosenberg scale ([20](#_ENREF_20)) | ● |  |  |  |  |
| General Self-Efficacy Scale ([21](#_ENREF_21)) | ● |  |  |  |  |
| Loneliness - De Jong Gierveld Scale ([22](#_ENREF_22)) |  | **●** |  |  |  |
| Loneliness single item |  | **●** |  |  |  |
| Stigma ([23](#_ENREF_23)) | **●** |  |  |  |  |
| Philadelphia Geriatric Center Morale Scale ([24](#_ENREF_24)) | **●** |  |  |  |  |
| the Yale Single question screen ([25](#_ENREF_25)) |  | **●** |  |  |  |
| Geriatric Depression Scale ([26](#_ENREF_26)) |  | **●** |  |  |  |
| **PHYSICAL FITNESS AND HEALTH** |  |  |  |  |  |
| Falling in last year ([27](#_ENREF_27)) |  | **●** |  | ● | ● |
| Sensory impairment – eyesight ([27](#_ENREF_27)) |  | **●** |  |  |  |
| Sensory impairment – hearing ([27](#_ENREF_27)) |  | **●** |  |  |  |
| Sleep quality ([28](#_ENREF_28), [29](#_ENREF_29)) | ● |  | ● |  |  |
| Changes in sense of taste ([30](#_ENREF_30)) |  | **●** |  |  |  |
| The short nutritional assessment questionnaire - single item ([31](#_ENREF_31)) |  |  |  | ● | ● |
| Mini-Nutritional Assessment ([32](#_ENREF_32)) |  |  |  | ● | ● |
| Alcohol | ● |  |  |  |  |
| Smoking | ● |  |  |  |  |
| Subjective health rating ([33-35](#_ENREF_33)) |  | **●** |  |  |  |
| Subjective memory |  | **●** |  |  |  |
| **RELATIONSHIP WITH CARER** |  |  |  |  |  |
| Nature of relationship with carer |  | **●** |  |  |  |
| Past relationship with carer (PAI) ([36](#_ENREF_36)) | ● |  |  |  |  |
| Current relationship with carer (PAI) ([36](#_ENREF_36)) |  | **●** |  |  |  |
| **MANAGING EVERYDAY LIFE WITH DEMENTIA** |  |  |  |  |  |
| Dependence Scale ([37](#_ENREF_37)) |  |  |  | ● | ● |
| Mini–Mental State Examination ([38](#_ENREF_38)) |  | **●** |  |  |  |
| Test for Severe Impairment ([39](#_ENREF_39)) |  | **●** |  |  |  |
| Neuropsychiatric symptoms (NPI) ([40](#_ENREF_40)) |  |  |  | ● | ● |
| The Global Deterioration Scale ([41](#_ENREF_41)) |  | **●** |  |  |  |
| Functional Assessment Staging ([42](#_ENREF_42), [43](#_ENREF_43)) |  | **●** |  |  |  |
| **RESOURCE INPUTS** |  |  |  |  |  |
| Health conditions completed with carer (where available) ([44](#_ENREF_44), [45](#_ENREF_45)) |  | **●** |  |  | **●** |
| CSRI completed with carer (where available): ([46](#_ENREF_46)) |  |  |  |  |  |
| CSRI hospital services |  | **●** |  |  |  |
| CSRI GP services |  | **●** |  |  |  |
| CSRI community healthcare services |  | **●** |  |  |  |
| CSRI home care and support services |  | **●** |  |  |  |
| CSRI community support services |  | **●** |  |  |  |
| CSRI residential care services |  | **●** |  |  |  |
| CSRI assistive technologies | **●** |  |  |  |  |
| CSRI informal care and support from family and friends |  | **●** |  |  |  |
| CSRI medication |  | **●** |  |  | **●** |
| Accommodation - satisfaction |  |  | ● |  |  |
| Accommodation (costs/type of residence) ([46](#_ENREF_46)) |  | **●** |  | **●** |  |
| **DESCRIPTIVE INFORMATION ABOUT INTERACTION WITH SERVICES** |  |  |  | ● |  |
| **FUTURE CARE NEEDS AND PLANNIING** |  | ● |  |  |  |
| **DIGNITY AND RESPECT** |  |  |  |  |  |
| Family treat with dignity and respect | ● |  | ● |  |  |
| Other people treat with dignity and respect | ● |  | ● |  |  |
| **ADJUSTMENT** |  |  |  |  |  |
| Representations and Adjustment to Dementia Index ([47](#_ENREF_47)) | ● |  | ● |  |  |
| **MAIN LIVING WELL MEASURES** |  |  |  |  |  |
| The Quality of Life-AD ([48](#_ENREF_48)) |  | **●** |  | **●** | **●** |
| Satisfaction with Life Scale ([49](#_ENREF_49)) |  | **●** |  | ● | ● |
| WHO5 well-being index ([50](#_ENREF_50)) |  | **●** |  | ● | ● |
| **OTHER LIVING WELL MEASURES** |  |  |  |  |  |
| Health state EQ5D ([51](#_ENREF_51)) |  | **●** |  |  |  |
| EQ5D visual analogue rating ([51](#_ENREF_51)) |  | ● |  |  |  |
| The Activity and Affect Indicators of Quality of Life - AAIQOL frequency of activities informant rating ([52](#_ENREF_52)) |  |  |  | ● | ● |
| AAIQOL expression of positive emotions - informant rating ([52](#_ENREF_52)) |  |  |  | ● | ● |
| Life is meaningful to me ([53](#_ENREF_53)) |  | **●** |  |  |  |
| Open ended questions focusing on living well |  | **●** |  |  |  |
| Open ended question: Is there anything else you’d like to tell us | **●** | **●** |  |  |  |

**Table 2.** Questions about carer

| **SOURCE OF INFORMATION** | **Relative/friend** | | **Member of care staff** |
| --- | --- | --- | --- |
| **TIME OF DATA COLLECTION** | **T4** | | **T4** |
| **MEASURES** | **v1** | **v2** | **v2** |
| **PERSONAL CHARACTERISTICS** |  |  |  |
| Date of birth |  | ● | ● |
| Country of birth (only asked if carer is new to the study) |  | ● |  |
| Location | ● | ● |  |
| Main language (only asked if carer is new to the study) |  | ● | ● |
| Gender |  | ● | ● |
| Ethnicity (only asked if carer is new to the study) | ● |  | ● |
| Marital status |  | ● |  |
| Sexual orientation (only asked if carer is new to the study) | ● |  |  |
| Number of children | ● |  |  |
| Religion (only asked if carer is new to the study) | ● |  |  |
| Accommodation (only asked if carer is new to the study) |  | ● |  |
| Life events ([54](#_ENREF_54)) |  | ● |  |
| **SOCIAL LOCATION** |  |  |  |
| Occupational status |  | ● |  |
| Social comparison | ● |  |  |
| The MacArthur Scale of Subjective Social Status - position on societal ladder ([3](#_ENREF_3)) |  | ● |  |
| The MacArthur Scale of Subjective Social Status - position on community ladder ([3](#_ENREF_3)) |  | ● |  |
| **CAPITALS ASSETS AND RESOURCES** |  |  |  |
| *Social capital - personal relations, social network support, civic engagement, trust and co-operative norms* |  |  |  |
| Personal relations – Office for National Statistics ([33](#_ENREF_33)) | ● |  |  |
| Social networks and support - Lubben scale ([4](#_ENREF_4), [5](#_ENREF_5)) |  | ● |  |
| Social networks and support - frequency of contact | ● |  |  |
| Social networks and support - satisfaction |  | ● |  |
| Willingness of people in neighbourhood to provide help ([55](#_ENREF_55)) | ● |  |  |
| Social participation - civic engagement (Office for National Statistics) ([6](#_ENREF_6)) | ● |  |  |
| Social participation - attendance at meetings of social and community groups (Office for National Statistics) ([6](#_ENREF_6)) | ● |  |  |
| *Cultural capital - institutionalised (education); incorporated (cultural participation)* |  |  |  |
| Education (only asked if carer is new to the study) | ● |  |  |
| Cultural capital - frequency and diversity of cultural activity ([56](#_ENREF_56)) | ● |  |  |
| *Other* |  |  |  |
| Activity engagement - social and cognitive activity ([8-10](#_ENREF_8)) | ● |  |  |
| Whether live in dementia-friendly community ([57](#_ENREF_57)) |  | ● |  |
| **ENVIRONMENT** |  |  |  |
| Neighbourhood - satisfaction with neighbourhood |  | ● |  |
| **PSYCHOLOGICAL CHARACTERISTICS AND PSYCHOLOGICAL HEALTH** |  |  |  |
| Personality - 5 dimensions ([13](#_ENREF_13)) | ● |  |  |
| Spirituality ([15-17](#_ENREF_15)) | ● |  |  |
| Optimism - Life Orientation Test – single item ([18](#_ENREF_18)) |  | ● |  |
| Self-esteem - Rosenberg scale ([20](#_ENREF_20)) | ● |  |  |
| Self-esteem - single item ([19](#_ENREF_19)) |  | ● |  |
| Generalized Self-efficacy Scale ([19](#_ENREF_19)) | ● |  |  |
| Loneliness - De Jong Gierveld Scale ([22](#_ENREF_22)) |  | ● |  |
| The Center for Epidemiologic Studies Depression Scale-Revised Short Form ([58](#_ENREF_58)) |  | ● |  |
| **PHYSICAL FITNESS AND HEALTH** |  |  |  |
| Falling in last year ([27](#_ENREF_27)) | ● |  |  |
| Medications | ● |  |  |
| Sensory impairment – eyesight ([27](#_ENREF_27)) |  | ● |  |
| Sensory impairment – hearing ([27](#_ENREF_27)) |  | ● |  |
| Alcohol | ● |  |  |
| Smoking | ● |  |  |
| Subjective health rating ([33-35](#_ENREF_33)) |  | ● |  |
| Health conditions |  | ● |  |
| **RELATIONSHIP WITH CARE RECIPIENT** |  |  |  |
| Relationship with care recipient |  | ● |  |
| Current relationship with participant (PAI) ([36](#_ENREF_36)) |  | ● |  |
| Past relationship with participant (PAI) ([36](#_ENREF_36)) | ● |  |  |
| **DESCRIPTIVE INFORMATION ABOUT CONTACT WITH SERVICES** |  |  |  |
| Information or educational materials, participation in support groups | ● |  |  |
| **INTERNET USE** |  |  |  |
| Access to the internet at home | ● |  |  |
| Use of the internet to assist or support relative/friend with dementia | ● |  |  |
| **EXPERIENCING CAREGIVING** |  |  |  |
| Information on caregiving |  | ● |  |
| Positive aspects of caregiving ([59](#_ENREF_59)) |  | ● |  |
| Caregiving Competence Scale ([60](#_ENREF_60)) |  | ● |  |
| Coping single item - taken from COPE Index ([61](#_ENREF_61)) |  | ● |  |
| Coping - management of meaning (the positive comparisons and reduction of expectations sub-scales) ([62](#_ENREF_62)) |  | ● |  |
| Role Captivity ([62](#_ENREF_62)) |  | ● |  |
| Relative/friend left unsupervised |  | ● |  |
| Relatives Stress Scale - total score ([63](#_ENREF_63)) |  | ● |  |
| SIDECAR questionnaire |  | ● |  |
| **MAIN LIVING WELL MEASURES** |  |  |  |
| The World Health Organization Quality of Life (WHOQOL)-BREF instrument ([64](#_ENREF_64)) |  | ● |  |
| Satisfaction with Life ([49](#_ENREF_49)) |  | ● |  |
| WHO5 well-being index ([50](#_ENREF_50)) |  | ● |  |
| **OTHER LIVING WELL MEASURES** |  |  |  |
| Health state EQ5D ([51](#_ENREF_51)) |  | ● |  |
| EQ5D visual analogue rating ([51](#_ENREF_51)) |  | ● |  |
| **RELATIVE/FRIEND IN A CARE HOME** |  | ● |  |
| Open ended questions for relatives/friends |  | ● |  |
| Open ended question: Is there anything else you’d like to tell us | ● | ● | ● |
| **SPECIFIC QUESTIONS ABOUT PAID CARER’SBACKGROUND** |  |  | ● |

**References**

1. CFAS Wales questionnaire [23/01/2018]. Available from: <http://www.cfas.ac.uk/files/2015/07/QuestionnairePart1English.pdf>.

2. Connell CM, Janevic MR, Solway E, McLaughlin SJ. Are pets a source of support or added burden for married couples facing dementia? Journal of Applied Gerontology. 2007;26(5):472-85.

3. Adler NE, Epel ES, Castellazzo G, Ickovics JR. Relationship of subjective and objective social status with psychological and physiological functioning: preliminary data in healthy white women. Health Psychology. 2000;19(6):586-92.

4. Lubben J, Blozik E, Gillmann G, Iliffe S, von Renteln Kruse W, Beck JC, et al. Performance of an abbreviated version of the Lubben Social Network Scale among three European community-dwelling older adult populations. The Gerontologist. 2006;46(4):503-13.

5. Lubben J, Gironda M, editors. Centrality of social ties to the health and well-being of older adults. New York, NY: Springer; 2003.

6. Office for National Statistics. Harmonised concepts and questions for social data sources, secondary standards. Social capital. Titchfield, UK: Office for National Statistics; 2008.

7. Bridges S, Doyle M, Fuller E, Knott C, Mindell J, Moody A, et al. Health Survey for England, 2012. Volume 2: Methods and Documentation. London: Health and Social Care Information Centre; 2013.

8. the Cognitive Function and Ageing Study (CFAS) [Available from: <http://www.cfas.ac.uk/>.

9. Cognitive function & Aging study, CFAS II [Available from: <http://www.cfas.ac.uk/cfas-ii/>.

10. Valenzuela M, Brayne C, Sachdev P, Wilcock G, Matthews F, Medical Research Council Cognitive Function and Ageing Study. Cognitive lifestyle and long-term risk of dementia and survival after diagnosis in a multicenter population-based cohort. Am J Epidemiol. 2011;173(9):1004-12.

11. James BD, Boyle PA, Buchman AS, Barnes LL, Bennett DA. Life space and risk of Alzheimer disease, mild cognitive impairment, and cognitive decline in old age. The American Journal of Geriatric Psychiatry. 2011;19(11):961-9.

12. Welsh Government. National Survey for Wales, 2012-2013. Cardiff: Welsh Government 2013.

13. Donnellan MB, Oswald FL, Baird BM, Lucas RE. The mini-IPIP scales: tiny-yet-effective measures of the Big Five factors of personality. Psychol Assess. 2006;18(2):192-203.

14. Loewenthal KM, MacLeod AK, Cinnirella M. Are women more religious than men? Gender differences in religious activity among different religious groups in the UK. Personality and Individual Differences. 2002;32(1):133-9.

15. King M, Speck P, Thomas A. The royal free interview for spiritual and religious beliefs: development and validation of a self-report version. Psychol Med. 2001;31(6):1015-23.

16. National Centre for Social Research. Scottish Social Attitudes Survey, 2001. Colchester, Essex: UK Data Archive; 2001.

17. National Centre for Social Research. British Social Attitudes Survey, 2008. Colchester, Essex: UK Data Archive; 2008.

18. Scheier MF, Carver CS, Bridges MW. Distinguishing optimism from neuroticism (and trait anxiety, self-mastery, and self-esteem): a reevaluation of the Life Orientation Test. J Pers Soc Psychol. 1994;67(6):1063-78.

19. Robins RW, Hendin HM, Trzesniewski KH. Measuring global self-esteem: construct validation of a single-item measure and the Rosenberg self-esteem scale. Personality and Social Psychology Bulletin. 2001;27(2):151-61.

20. Rosenberg M. Society and the adolescent self-image. Princeton, NJ: Princeton University Press; 1965.

21. Schwarzer R, Jerusalem M. Generalized Self-Efficacy Scale. In: Weinman J, Wright S, Johnston M, editors. Measures in health psychology: a user’s portfolio Causal and control beliefs. Windsor, UK: NFER-NELSON; 1995. p. 35-7.

22. De Jong Gierveld J, Tilburg TV. A 6-item scale for overall, emotional, and social loneliness confirmatory tests on survey data. Research on Aging. 2006;28(5):582-98.

23. Fife BL, Wright ER. The dimensionality of stigma: a comparison of its impact on the self of persons with HIV/AIDS and cancer. J Health Soc Behav. 2000;41(1):50-67.

24. Lawton MP. The Philadelphia Geriatric Center Morale Scale: a revision. J Gerontol. 1975;30(1):85-9.

25. Mahoney J, Drinka TJ, Abler R, Gunter-Hunt G, Matthews C, Gravenstein S, et al. Screening for depression: single question versus GDS. J Am Geriatr Soc. 1994;42(9):1006-8.

26. Almeida OP, Almeida SA. Short versions of the Geriatric Depression Scale: a study of their validity for the diagnosis of a major depressive episode according to ICD-10 and DSM-IV. International Journal of Geriatric Psychiatry. 1999;14(10):858-65.

27. English Longitudinal study of Ageing (Elsa). [Available from: <https://www.elsa-project.ac.uk/>.

28. Buysse DJ, Reynolds CF, 3rd, Monk TH, Berman SR, Kupfer DJ. The Pittsburgh Sleep Quality Index: a new instrument for psychiatric practice and research. Psychiatry research. 1989;28(2):193-213.

29. Clare L, Whitaker CJ, Craik FI, Bialystok E, Martyr A, Martin-Forbes PA, et al. Bilingualism, executive control, and age at diagnosis among people with early-stage Alzheimer's disease in Wales. Journal of neuropsychology. 2016;10(2):163-85.

30. Heald AE, Pieper CF, Schiffman SS. Taste and smell complaints in HIV-infected patients. AIDS. 1998;12(13):1667-74.

31. Wilson MM, Thomas DR, Rubenstein LZ, Chibnall JT, Anderson S, Baxi A, et al. Appetite assessment: simple appetite questionnaire predicts weight loss in community-dwelling adults and nursing home residents. The American Journal of Clinical Nutrition. 2005;82(5):1074-81.

32. Guigoz Y, Vellas B, Garry PJ. Mini Nutritional Assessment: a practical assessment tool for grading the nutritional state of elderly patients. Facts and Research in Gerontology. 1994;4((Supplement 2)):15-59.

33. Office for National Statistics [Available from: <https://www.ons.gov.uk/>.

34. Bowling A. Just one question: if one question works, why ask several? Journal of Epidemiology and Community Health. 2005;59(5):342-5.

35. Ware JE KM, Keller SK. . SF-36® Physical and Mental Health Summary Scales: A User's Manual. Boston, MA: The Health Institute; 1994.

36. Bengtson VL, Schrader SS. Parent-child relations. In: Mangon DJ, Peterson WA, editors. Research instruments in social gerontology: Social roles and social participation. 2. Minnesota: University of Minnesota Press; 1982. p. 115-85.

37. Brickman AM, Riba A, Bell K, Marder K, Albert M, Brandt J, et al. Longitudinal assessment of patient dependence in Alzheimer disease. Arch Neurol. 2002;59(8):1304-8.

38. Folstein MF, Folstein SE, McHugh PR. “Mini-mental state”. A practical method for grading the cognitive state of patients for the clinician. J Psychiatr Res. 1975;12(3):189-98.

39. Albert M, Cohen C. The Test for Severe Impairment: an instrument for the assessment of patients with severe cognitive dysfunction. J Am Geriatr Soc. 1992;40(5):449-53.

40. Kaufer DI, Cummings JL, Ketchel P, Smith V, MacMillan A, Shelley T, et al. Validation of the NPI-Q, a brief clinical form of the Neuropsychiatric Inventory. J Neuropsychiatry Clin Neurosci. 2000;12(2):233-9.

41. Reisberg B, Ferris SH, de Leon MJ, Crook T. The Global Deterioration Scale for assessment of primary degenerative dementia. Am J Psychiatry. 1982;139(9):1136-9.

42. Reisberg B. Functional Assessment Staging (FAST). Psychopharmacol Bull. 1988;24(4):653-9.

43. Sclan SG, Reisberg B. Functional assessment staging (FAST) in Alzheimer's disease: reliability, validity, and ordinality. International Psychogeriatrics. 1992;4 Suppl 1:55-69.

44. Charlson ME, Charlson RE, Peterson JC, Marinopoulos SS, Briggs WM, Hollenberg JP. The Charlson comorbidity index is adapted to predict costs of chronic disease in primary care patients. Journal of Clinical Epidemiology. 2008;61(12):1234-40.

45. Charlson ME, Pompei P, Ales KL, MacKenzie CR. A new method of classifying prognostic comorbidity in longitudinal studies: development and validation. Journal of Chronic Diseases. 1987;40(5):373-83.

46. Beecham J, Knapp M. Costing psychiatric interventions. In: Thornicroft G, Brewin C, Wing J, editors. Measuring mental health needs. 2 ed. London: Gaskell; 2001. p. 203-27.

47. Quinn C, Morris RG, Clare L. Beliefs About Dementia: Development and Validation of the Representations and Adjustment to Dementia Index (RADIX). The American journal of geriatric psychiatry : official journal of the American Association for Geriatric Psychiatry. 2018;26(6):680-9.

48. Logsdon RG, Gibbons LE, McCurry SM, Teri L. Quality of life in Alzheimer's disease: patient and caregiver reports. Journal of Mental Health and Aging. 1999;5(1):21-32.

49. Diener E, Emmons RA, Larsen RJ, Griffin S. The Satisfaction With Life Scale. J Pers Assess. 1985;49(1):71-5.

50. Bech P. Measuring the dimension of psychological general well-being by the WHO-5. Quality of Life Newsletter. 2004;32:15-6.

51. The EuroQol Group. EuroQol - a new facility for the measurement of health-related quality of life. Health Policy. 1990;16(3):199-208.

52. Albert SM, Del Castillo-Castaneda C, Sano M, Jacobs DM, Marder K, Bell K, et al. Quality of life in patients with Alzheimer's disease as reported by patient proxies. J Am Geriatr Soc. 1996;44(11):1342-7.

53. Surveys using the 4 Office for National Statistics personal well-being questions [Available from: <https://www.ons.gov.uk/peoplepopulationandcommunity/wellbeing/methodologies/surveysusingthe4officefornationalstatisticspersonalwellbeingquestions>.

54. Holmes TH, Rahe RH. The Social Readjustment Rating Scale. J Psychosom Res. 1967;11(2):213-8.

55. Understanding Society. The UK household longitudinal study [Available from: <https://www.understandingsociety.ac.uk/>.

56. Thomson K. Cultural capital and social exclusion survey: technical report. London: National Centre for Social Research; 2004.

57. Society As. Foundation criteria for the dementia-friendly communities recognition process. 2014-2015.

58. Eaton WW, Smith C, Ybarra M, Muntaner C, Tien A. Center for Epidemiologic Studies Depression Scale: review and revision (CESD and CESD-R). In: Maruish ME, editor. The Use of Psychological Testing for Treatment Planning and Outcomes Assessment. Volume 3: Instruments for Adults. 3rd ed. Mahwah, NJ: Lawrence Erlbaum; 2004. p. 363-77.

59. Tarlow BJ, Wisniewski SR, Belle SH, Rubert M, Ory MG, Gallagher-Thompson D. Positive Aspects of Caregiving contributions of the REACH project to the development of new measures for Alzheimer’s caregiving. Research on Aging. 2004;26(4):429-53.

60. Robertson SM, Zarit SH, Duncan LG, Rovine MJ, Femia EE. Family caregivers’ patterns of positive and negative affect. Family Relations. 2007;56(1):12-23.

61. McKee KJ, Philp I, Lamura G, Prouskas C, Oberg B, Krevers B, et al. The COPE index--a first stage assessment of negative impact, positive value and quality of support of caregiving in informal carers of older people. Aging Ment Health. 2003;7(1):39-52.

62. Pearlin LI, Mullan JT, Semple SJ, Skaff MM. Caregiving and the stress process: an overview of concepts and their measures. The Gerontologist. 1990;30(5):583-94.

63. Greene JG, Smith R, Gardiner M, Timbury GC. Measuring behavioural disturbance of elderly demented patients in the community and its effects on relatives: a factor analytic study. Age Ageing. 1982;11(2):121-6.

64. Skevington SM, Lotfy M, O'Connell KA, Group W. The World Health Organization's WHOQOL-BREF quality of life assessment: psychometric properties and results of the international field trial. A report from the WHOQOL group. Quality of Life Research. 2004;13(2):299-310.
